# ‘I struggle to feel normal’: phenomenological analysis of experience with living in crises resettlement camps in Nigeria

**DOI:** 10.1093/inthealth/ihaf065

**Published:** 2025-06-24

**Authors:** Promise Nmesomachi Timothy, Uchenna Frank Imo, Chimankpam Kingsley Ogbonna, Abuo James, Temidayo Akinreni, Mfonobong Akpandem, Miracle Nwadiche, Precious Azubuike

**Affiliations:** Department of Human Anatomy, University of Uyo, Uyo, 1017, Nigeria; Department of Public Health, College of Medical Sciences, University of Calabar, Calabar, 1115, Nigeria; Department of Public Health, College of Medical Sciences, University of Calabar, Calabar, 1115, Nigeria; Department of Public Health, College of Medical Sciences, University of Calabar, Calabar, 1115, Nigeria; Heidelberg Institute of Global Health, Ruprecht-Karls Universität Heidelberg, Heidelberg 69120, Germany; Department of Public Health, College of Medical Sciences, University of Calabar, Calabar, 1115, Nigeria; Department of Psychology, Ebonyi State University, Abakaliki 481101, Nigeria; Department of Public Health, College of Medical Sciences, University of Calabar, Calabar, 1115, Nigeria

**Keywords:** humanitarian crises, internally displaced persons, psychosocial support, qualitative research, trauma

## Abstract

**Background:**

Nigeria has experienced several humanitarian crises and displacement over the years. The physical and psychological impact of these crises has been severe, with many experiencing trauma and stress-related disorders within internally displaced persons camps. To develop interventions that address the needs of settlers in camps, a comprehensive understanding of their experiences is essential.

**Methods:**

Our qualitative study employed phenomenological analysis to explore the lived experiences of victims of humanitarian crises within internally displaced persons camps. Purposive sampling was used to recruit participants for the focus group discussions, and we employed snowballing to recruit participants for the in-depth interviews.

**Results:**

Four themes and 10 subthemes emerged from the analysis. Participants’ experiences were laced with harsh physical living conditions, including deteriorated and leaking roofs in camps, inadequate water, sanitation and hygiene facilities and limited autonomy and self-sustenance. The inability to connect with relatives outside the camps fostered feelings of isolation, hopelessness and anxiety.

**Conclusions:**

Our study emphasizes the importance of establishing livable conditions for displaced persons living in camps and other resettlement settings, while fostering social connections with external communities. This connection may enhance their hopes for a normal life, build resilience and facilitate their social integration back into society.

## Introduction

Global humanitarian crises, ranging from natural disasters to armed conflicts and inter-tribal clashes, have led to the displacement of >100 million people over the last decade, leading to growing concerns regarding trauma, mental health and reintegration.^1^ These crises have long-term devastating effects on the populations affected, especially internally displaced persons (IDPs), whose psychological and social needs are often neglected in response programs. Hence, the WHO emphasizes the need to understand lived experiences and provide tailored mental health and psychosocial support to people in crisis-affected settings.^2^

Nigeria has experienced several humanitarian crises over the years, including conflict, natural disasters and displacement. These crises have resulted in the displacement of millions of people, leaving them without necessities, traumatized and vulnerable to psychosocial problems.^3,4^ In Nigeria, according to the Internally Displacement Monitoring Centre, >290 000 people were displaced in 2023 due to crises, doubling the figure recorded in 2022.^5^ The psychosocial effects of these crises continue to linger long after the physical damages have been repaired, making it difficult for victims to reintegrate into society.^6^

One of the most significant humanitarian crises in recent times in Nigeria is the Boko Haram insurgency, which began in 2009.^7^ The conflict has resulted in the displacement of >3 million people, with many forced to flee their homes and seek refuge in neighboring countries as of 2023.^5,8^ In addition to the Boko Haram conflict, Nigeria has also experienced several natural disasters, including floods, droughts and epidemics such as Lassa fever, cholera and the Ebola outbreak, all of which have exacerbated the challenges faced by displaced populations.^9^ These disasters have resulted in the displacement of millions of people, leaving them without basic necessities.^10^

The immediate impact of displacement includes loss of homes and shelter, loss of thousands of lives, livelihoods, food, water and access to essential services such as healthcare and education.^11^ The impact further transcends to abduction of women and children leading to rape and forced marriages with Boko Haram members.^11^ Previous research has shown that IDPs face severe hardship and challenges, including poverty and substandard livening conditions,^12^ and are thereby vulnerable to preventable infections such as malaria, acute respiratory disorder or non-communicable diseases such as cardiovascular disease or hypertension.^13,14^ Furthermore, these experiences have left many traumatized, with many experiencing trauma, anxiety disorders and stress-related disorders.^15,16^

The impact of humanitarian crises can have long-term effects on individuals and communities.^17^ Studies have shown that individuals who have experienced conflict or displacement are more likely to experience depression, anxiety and post-traumatic stress disorder (PTSD).^18,19^ They may also experience feelings of hopelessness and helplessness, which can lead to self-harm and suicide.^20^ Individuals who have experienced trauma and stress-related disorders may find it difficult to reintegrate into society, leading to social isolation and marginalization.^21^

Nigeria has taken steps to address these issues through national mental health policies, IDP camp programs and support from partners like the WHO and UNICEF. However, implementation is inconsistent, and services remain centralized and underfunded, as the Nigerian health system is ill-equipped to handle the psychosocial needs of this population, and there are few resources dedicated to the provision of support services.^22,23^ Community-level psychosocial support is often lacking, especially in remote camps where reintegration and trauma recovery needs are most acute.^22^ This has resulted in increased incidences of mental health problems, low self-esteem and a reluctance to reintegrate into society among victims.^24,25^ For instance, when abducted women and girls are released, the communities tend to label them ‘Boko Haram Wives’ or the local word ‘*annoba’*, which means epidemics, as members of communities fear that they have been indoctrinated into the tenets of captivity.^10,22^ Isolated and spurned, these women and girls face dire poverty and some are even forced into prostitution to feed their children.^26^ This can result in a breakdown of social cohesion, which can further exacerbate the effects of the humanitarian crisis. While reports acknowledge these challenges, there is limited empirical evidence, especially qualitative that highlights IDPs' lived realities and voices.

The lack of adequate psychosocial support services for victims of humanitarian crises in Nigeria has further compounded the problem. The Nigerian health system is ill-equipped to handle the psychosocial needs of this population, and there are few resources dedicated to the provision of support services, resulting in increased incidences of anxiety, depression, PTSD and emotional distress, as well as low self-esteem and a reluctance to reintegrate into society among victims.^22,23^

However, although these concerns and the importance of tailor-made support services in addressing the impact of humanitarian crises have been acknowledged by national and international actors,^27^ few studies have employed in-depth qualitative methods to explore how displaced persons experience these intersecting challenges in Nigeria.^28–30^ Understanding these lived experiences of people living in settings of displacement—especially the burdens, barriers to reintegration and social stigma—can inform evidence-based interventions toward bridging current program and intervention gaps.^31,32^ This study aimed to explore these lived experiences in depth, giving voice to IDPs and emphasizing the critical areas where support and intervention are most urgently needed.

## Methods

### Study design

We adopted a qualitative method for our study, leveraging the constructivist epistemology, which is grounded in the belief that there is no single ‘truth’.^33–35^ Additionally, this study adopted phenomenology analysis to assess the lived experiences of victims of humanitarian crises. This approach allowed participants to convey their insights and experiences in a way that reflects their unique perspectives and contexts.^36,37^

### Study location/area

The study was conducted in Bakassi Local Government Area in Cross River State, Nigeria, a region that hosts the Bakassi IDP camp, one of the nation's largest IDP resettlements due to the country's ongoing humanitarian challenges.

### Study population

This study focused on individuals affected by humanitarian crises living in Bakassi IDP Camp, Bakassi Local Government Area of Cross River State, Nigeria. The source population included all residents of the camp who were displaced due to conflicts or disasters. Participants were included if they were aged ≥18 y, had resided in the camp for at least 6 mo and were willing to share their experiences. We excluded children aged <18 y due to ethical considerations, including the need for parental consent, which was difficult to obtain in a displaced setting. Also, the depth of the topics explored, such as lived experiences and reintegration, required a level of cognitive maturity that is more common in adults. Lastly, including children would have required additional psychosocial support measures, which were not available in the study setting. Participants were selected based on their direct lived experiences of displacement, ensuring a comprehensive understanding of the challenges they faced in camp.

### Sample size/procedure

Purposive sampling was used to select adult participants (aged ≥18 y) in the study. Four focus group discussions (FGDs) were conducted in the camp, with eight adults per FGD. Additionally, eight respondents participated in the in-depth interview (IDI) sessions. For the IDIs, a snowball technique was used, whereby participants referred us to other potential respondents who could provide valuable insights. We used purposive sampling to recruit participants for the FGDs to ensure diverse group representation across age, gender and other relevant characteristics. For the IDIs, most of the potential participants, especially those with first-hand sensitive experiences, were not easily accessible through our formal channels. Snowball sampling allowed us to identify and reach these information-rich participants who otherwise were difficult to reach, especially in the camp setting where trust and social networks are essential for access. Participants in the initial IDIs referred to others they knew who shared similar experiences and characteristics; hence, the depth and diversity of perspectives we gathered during the IDI sessions were enhanced. We leveraged the principle of saturation or redundancy of information as recommended by Polit and Beck for the data collection as a widely accepted standard in qualitative research.^38^ Consequently, we conducted interviews and discussions until saturation was reached, meaning that the study's objectives were met, or when participants began to provide repetitive information.

### Tools/procedure for data collection

Data were collected using pretested semistructured guides for the FGD and IDI data collection. Prior to data collection, ethical clearance was obtained from the Research and Ethics Committee of the Cross River State Ministry of Health (approval number: CRS/MH/HREC/024/283). Also, officers in charge of the camp were contacted to facilitate data collection among women in IDP households. Both written and verbal informed consent were obtained from all participants prior to their involvement in the study. Participants were clearly informed about the purpose of the study, their voluntary participation, the right to withdraw at any time without consequences and the measures taken to ensure confidentiality and data protection.

Three trained field research assistants were recruited and trained for the data collection with the supervision of the principal investigator and co-authors. The data were collected over 2 mo of the project in the communities. The FGDs and IDIs were recorded using an audio recorder. The FGDs and IDIs were conducted in Pidgin English and Efik/Ibibio languages, which are the local languages used in the camp and the mother tongues of the principal investigator and co-authors.

### Data analysis

The transcripts from the FGDs and IDIs were transcribed verbatim to reflect and maintain participants’ words in the original meaning and context. Two independent researchers individually reviewed and vetted the transcript for quality control before data analysis. Inductive and deductive (hybrid) thematic analysis approaches were used for the analysis with a predesigned codebook generated from the study instruments, as well as in-depth reading of the transcripts. Before data analysis, researchers familiarized themselves with the transcripts through iterative readings to generate initial common themes and subthemes from the transcript. The initial themes and subthemes formed the basis for the codebook. The codebook was iteratively revised leveraging existing literature.^3,31^ The data were imported to the NVivo (version 14) application for appropriate codes, nodes and themes generation. As new themes emerged during the analysis, we iteratively incorporated them into the codebook and applied them systematically to the entire dataset, refining the thematic framework to capture the data's contents.

## Results

Table 1 shows the sociodemographic characteristics of participants in our study. One-half of the participants in the FGDs and IDIs were aged 31–40 y. In terms of marital status, more than two-thirds (67%) of our participants in the FGDs and IDIs were single.

**Table 1.** Sociodemographic characteristics of participants (FGDs, n=32; IDIs, n=8)

**Characteristics**	**IDIs** **Frequency (%)**	**FGDs** **Frequency (%)**
**Age (y)**		
21–30	4 (50.0)	16 (50.0)
31–40	2 (25.0)	8 (25.0)
≥41	2 (25.0)	8 (25.0)
**Gender**		
Male	5 (62.5)	18 (56.3)
Female	3 (37.5)	14 (43.7)
**Marital status**		
Single	5 (67.0)	22 (67.0)
Married	2 (25.0)	8 (25.0)
Separated	1 (12.5)	4 (12.5)
**Education**		
No formal education	3 (37.5)	20 (62.5)
Primary	2 (25.0)	4 (12.5)
Secondary	3 (37.5)	8 (25.0)
**Current employment status**		
Unemployed	4 (50.0)	16 (50.0)
Business/tradesperson	1 (12.5)	8 (25.0)
Farming	2 (25.0)	4 (12.5)
Civil service	1 (12.5)	4 (12.5)

FGD: focus group discussion; IDI: in-depth interview.

### Summary of key themes and subthemes

**Figure 1.**
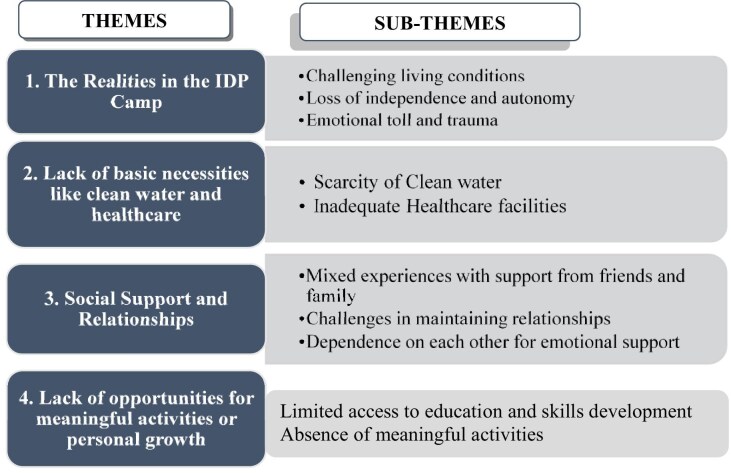
Summary of key themes and subthemes.

Four themes and 10 subthemes emerged from the analysis (Figure 1); life within the camp was marked by challenging living conditions, as well as by a loss of independence and autonomy, resulting in emotional toll and trauma. Respondents faced limited access to essential resources such as clean water and adequate healthcare, which further endangered their health and well-being. Social support systems within and outside the camp offer mixed experiences; while some encountered challenges in maintaining relationships, others depended on one another for emotional support. However, internal support networks among residents serve as critical emotional lifelines. Additionally, the camp environment offers limited opportunities for education, the acquisition of skills or meaningful engagement, resulting in feelings of stagnancy and hopelessness about the future.

### Realities in the IDP camp

The residents described life within the camp as being marked by several difficulties. They highlighted challenging physical living conditions, including a lack of privacy because of overcrowding and the loss of autonomy as they feel strongly that they have become dependent and reliant on survival aids. Discussants highlighted the emotional impact of displacement as profound, describing the transition as traumatic, with feelings of hopelessness and daily experiences of despair. Furthermore, the scarcity of clean water and adequate healthcare, which are basic necessities, intensifies their struggle to survive and thrive.

#### Challenging living conditions

Discussants highlighted the reality of daily life in the IDP camp as harsh, just as the initial trauma of displacement wears off. Noting the overcrowding in the camp, they expressed how they have begun to live without any personal space. The conditions of the living areas also exacerbated the unpleasant displacement experience, with rainfall causing more discomfort in the camp.

Beyond the physical hardships, participants expressed a strong sense of lost dignity and unfair treatment. Many felt their current living conditions, marked by overcrowding, lack of privacy and poor infrastructure, were not what they deserved. This sense of injustice was closely tied to the loss of independence, as they moved from self-reliance to full dependence on aid:

*To be honest, it shames me. We used to have our own houses and were managing our own lives, but now, it is only handouts we wait for, and we are managing in this kind of cramped environment. The whole thing about not being able to do things for myself again and depend on people for everything just pains me in the heart, and I can't help but feel bad about how things are now* (FGD Participant 2).

*Yes, living in this place is not easy at all. We are packed in here like sardines, and there is no space to even turn around. There is nothing to separate you from your neighbor, and the rain and sun beat us any which way here. This is not how life is supposed to be! You can imagine how it feels, lack of basic clean water, even privacy for sanitation and even quality healthcare, it can make someone unsure of what the next day holds* (IDI Participant 1).

*This condition we face here makes no sense! We deserve a better situation than this, but unfortunately, we're trapped and unable to escape or improve our circumstances. Sometimes, it feels like the government does not care, and when it rains here, the whole place becomes a mess. You can imagine the situation for us, already we are trying to manage the space here. This affects a person's sense of human and well-being* (FGD Participant 5).

#### Loss of independence and autonomy

The realities of the camp extend beyond physical hardships. The transition from self-sufficiency to dependence on aid dismantles the displaced individual's sense of autonomy and control. Participant narratives paint a picture of humiliation and shame as they navigate cramped living conditions and rely on external assistance for basic needs. This loss of control over their daily lives amplifies their vulnerability and erodes their sense of self-worth. For the residents, the reliance on aid, even for the most basic of necessities, serves as a constant reminder of their displacement and the limitations it imposes. The inability to provide for themselves and the lack of basic human dignity become heavy burdens for the displaced individuals within the IDP camp:

*This place makes it very hard to feel good about ourselves. We always need help from others, even for basic things. It's not right…* (FGD Participant 4).

*Life here is very difficult. People don't see us or treat us well. It makes us feel alone and unimportant. We just want to be treated like people and have what we need to survive* (FGD Participant 6).

*The whole thing about not being able to do things for myself again and depend on people for everything just pains me in the heart, and I can't help but feel bad about how things are now* (IDI Participant 2).

#### Emotional toll and trauma

The physical hardships and loss of autonomy within the IDP camp were only a part of the harsh reality. Discussants highlighted the profound emotional impact of displacement, describing the transition as traumatic with feelings of hopelessness and daily experiences of despair. Discussants shared their experiences with displacement, marked by anxiety, sense of insecurity, fear and, for some, depression, resulting from the grief of loss, including the loss of sources of livelihood. For some, the psychological impacts of displacement persisted, a perpetual reminder of the trauma they had faced. With suboptimal mental health support in the camp, the displaced individuals are left to navigate their emotional pain within the cramped and challenging environment of the IDP camp, where their struggles often remain hidden and unaddressed:

*The emotional burden of life in the camp should not be downplayed…Many of us grapple with intense feelings of sorrow, anxiety and despair, yet there is a scarcity of resources to address our mental well-being* (FGD Participant 16).

*I think you should know that the struggle to feel normal in this camp, and other camps extends beyond physical needs. Most of us grapple with a sense of isolation and worthlessness, further amplified by the lack of respect we experience. Life here is very difficult. People don't see us or treat us well. It makes us feel alone and unimportant* (FGD Participant 14).

*The loss of our former lives adds another layer of pain. This also has an impact on our self-worth. You should know these things. The harsh conditions in the camp have eroded our dignity and self-respect. We once had stable lives, homes and livelihoods, but now we rely on aid…It is sad to feel like you have been stripped of your autonomy and left incapable to decide what next you will do and how you want your life and future to be!* (IDI Participant 3).

### Lack of basic necessities such as clean water and healthcare

The fight for survival in the IDP camp transcends the emotional toll. Participants face relentless struggles to access basic resources essential for health and well-being. A recurring theme in participant narratives was the scarcity of clean water and inadequate healthcare facilities. Moreover, participants noted that the scarcity of basic necessities within the camp such as sanitation facilities, clean water and quality healthcare services fuels the struggles for them in the camp, causing anxiety, fear and raising concerns about hygiene and sanitation:

*On top of everything else, getting basic things like clean water to survive is a real problem in this camp…We wait in long lines just to collect water, and even then, it's not always clean. How can someone stay healthy when even the water we drink is uncertain? You should know that the scarcity of clean water is a breeding ground for illness, further jeopardizing our health* (FGD Participant 23).

*Access to medical attention here is limited, and this makes us feel exposed and unable to properly address our health concerns. Clean water and doctor help, everyone needs that. But here, it's very hard to get. We worry a lot about getting sick because the place to get help is not good* (FGD Participant 12).

*The healthcare here is not good at all. The facilities where they treat people are inadequate, and some of the people who work there don't even understand our illnesses. It's a big problem to see a doctor when you need one. Sometimes you wait for hours for your turn, and by the time you see the doctor, your sickness has gotten even worse* (IDI Participant 7).

### Social support and relationships

Despite the numerous difficulties in the IDP camp, participants find a potent source of strength in their social bonds. The shared struggles, friendships and collective support serve as a resilience factor, enabling individuals to navigate the challenges of displacement and envision a more promising tomorrow. However, discussants highlighted that their ability to maintain relationships with people outside the camp is limited, often leading to feelings of isolation from the broader community. They often experience isolation from the broader community, highlighting the importance of internal support systems within the camp. While these internal bonds provide a crucial lifeline, the yearning for connection with loved ones and the life they left behind persists.

#### Mixed experiences with support from friends and family

The importance of social support extends beyond the confines of the IDP camp. Participants often relied on connections with friends and family who reside outside the camp. These external relationships can be a vital lifeline, offering emotional and material assistance during a time of immense hardship. However, participants’ narratives reveal a varied experience, highlighting the challenges alongside the solace found in these connections:

*We are fortunate to have supportive friends and family, but it's not always straightforward. I am deeply thankful for the steadfast support of our loved ones. Regular communication, even if only online, provides a sense of reassurance that we are not isolated* (IDI Participant 4).

*You can imagine that sometimes, truth be told, supporting displaced individuals can also strain the resources of people that care for us, potentially leading to feelings of burden on both sides. They may not say it but our loved ones may grapple with feelings of guilt or shame related to their circumstances* (FGD Participant 11).

*There is definitely a time when it feels like people avoid interacting with us, especially those from outside the camp. However, support from family and friends is very important, but it's not always enough* (FGD Participant 7).

The fight for survival and a sense of normalcy within the IDP camp extends beyond physical limitations. Participants navigate a complex web of social connections, both within and outside the camp. While external support offers solace and assistance, it is often accompanied by its own set of challenges.

#### Challenges in maintaining relationships

Geographical separation emerged as a significant hurdle in the wake of displacement. Scattered families and friends across vast distances make physical visits infrequent or even impossible. The physical distance is exacerbated by the unpleasant conditions within the camp, in which cramped tents that lack privacy alongside the relentless stress of daily life create an environment that discourages meaningful communication among residents. Moreover, limited access to phones and unreliable internet connections restricts regular contact, leading to feelings of isolation and a disintegration of social connections:

*This whole situation in the camp makes us physically distant from our loved ones, and on top of that, transportation isn't always available to travel and see them. The displacement itself creates a physical barrier, and limited resources make overcoming it a constant challenge* (FGD Participant 18).

*The distance and the way things are in this camp make it hard to hold onto strong connections with our loved ones. How can someone possibly rebuild their life when they're unsure what the next day will bring?* (FGD Participant 4).

*Some people from outside the camp, especially those who don't live here, often don't want to engage with us. It's as if they're not comfortable because of our circumstances. Although this is just my feeling. But you know this feeling can make it even harder for us to maintain connections with loved ones* (IDI Participant 1).

The cumulative effect of these factors presents a bleak scenario for displaced individuals seeking to maintain social relationships. The physical distance, scarce resources and unpleasant living conditions converge to create a profound sense of isolation, further exacerbating the already daunting challenges they face.

#### Dependence on each other for emotional support

Despite struggles and uncertainties, participants reported that they are comforted in their shared experiences; they form strong bonds with one another and provide emotional support in times of need. Their shared struggle in the camp provides a common ground for them to bond and thrive. When challenges arise, individuals turn to each other for support, forming tight-knit networks that offer comfort, belonging and a sense of purpose. This shared connection becomes an immense source of resilience and hope, helping them cope with the challenges associated with displacement:

*Here in the camp, the way we manage to survive is through dependence and support. We lean on each other for strength and try to stay positive despite the challenges* (FGD Participant 2).

*Living alone here is hard. It's not easy to find people who know what we're feeling. But we help each other feel better and try to stay positive even though things are difficult* (FGD Participant 16).

Support within the camp goes beyond emotional comfort, as individuals share resources, skills and responsibilities, forming a network of mutual aid that strengthens collective well-being and empowers displaced individuals to navigate challenges and hold onto hope for a brighter future.

### Lack of opportunities for meaningful activities or personal growth

While the struggle for survival dominates daily life within the IDP camp, a deeper yearning emerges in participant narratives: the yearning for purpose and personal growth. The realities of the camp environment stifle opportunities for meaningful activities and pursuits that could foster a sense of fulfillment. This lack of stimulation and personal growth leads to feelings of being stuck, unmotivated and emotionally drained:

*For everyone else, it is normal to want to learn a skill, have a life and connect with people, being in this camp makes it feel like there is no more future, anyways, I keep looking out for opportunities to grow–But that is not the case with everyone else* (IDI Participant 5).

*Sometimes, it is as if people who are here do not have a life outside of the camp, or do not plan for it. There is a way the impact of being here can make a person feel their sense of purpose has been eroded. People here should also learn to build skills and develop capacities that contribute to meaningful engagement, and a sense of achievement* (IDI Participant 21).

## Discussion

Our study participants described their lived experiences as being marked by numerous challenges within the IDP camp. The daily experiences of IDPs are laced with harsh physical living conditions including deteriorated and leaking roofs in camps, lack of water, sanitation and hygiene facilities, as well as limited means of autonomy and self-sustenance. Key among these were the difficult physical living conditions stemming from the leaking roofs of buildings within the camp, which are worse when it rains; severe overcrowding that leads to a lack of privacy; the emotional toll and trauma; as well as the shortage of clean, safe water and healthcare. These align with previous studies conducted in Bangladesh and Nigeria among the displaced populace, which showed that IDP environments are often structurally compromised, creating unsafe and undignified living spaces.^39–42^ Such environments not only exacerbate daily hardships but also contribute to diminished autonomy, a reduced sense of personal space, psychological stress and physical health risks. The erosion of the physical living space coincides with a symbolic loss of self-worth and control, as many participants’ autonomy is undermined by their dependence on humanitarian aid and the lack of private or secure personal space.^43^

In addition to the physical and emotional challenges, the scarcity of basic necessities such as safe, clean water and inadequate healthcare facilities contributes to further struggles in the IDP camp. Participants in our study had to queue for a long time before collecting little or no water, which jeopardizes their health and further breeds infectious diseases and illness. Our findings suggest that this loss of agency is reinforced not only by the structural conditions but also by the scarcity of essential services, especially water and healthcare. Previous research in Africa revealed that limited access to essential services such as water, medications and healthcare facilities in the IDP camp significantly results in increased infectious and communicable diseases.^44,45^ When basic survival becomes a daily struggle, displaced individuals experience compounded helplessness and despair, reinforcing cycles of trauma. The inability to access clean water or timely medical assistance was a prominent dimension of the loss of autonomy, which reflects broader patterns observed in African and Middle Eastern IDP settings.^46,47^ Theoretically, this supports the concept of ‘structural violence’, a condition in which social structures inhibit individuals from meeting their basic needs and thus highlights the pertinent necessity for robust services and support in the IDP camp to address both the immediate needs and long-term health and psychosocial outcomes.^48,49^

Notably, these experiences such as a lack of basic amenities may vary across different contexts. Studies indicate that victims of humanitarian crises, including those in Nigeria and other West African countries, face diverse challenges.^50,51^ These variations are attributed to several factors including political instability, socioeconomic disparities and resource allocation.^50,51^ Additionally, the societal attitude, norms and sociocultural contexts of the populace toward IDPs may influence their coping strategies in various contexts.^3,52^ Previous research in different geographical contexts in Nigeria has also shown similar challenges in the IDP camp, including limited resources, a lack of integration and social acceptance, as well as a lack of opportunities for meaningful activities or personal growth.^53,54^ While the findings on their experiences align with the general spectrum, individual differences may stem from regional differences in sociocultural norms and acceptance.^14,55,56^ These disparities in reported experiences across different studies might stem from methodological differences, including study locations and data collection techniques.^57,58^ Hence, there is a need for tailored, IDP-led interventions to tackle the complex challenges facing IDPs in crisis areas.^58^

Hence, such environments not only exacerbate daily hardships but also contribute to diminished autonomy, sense of personal space and security. This loss of autonomy further contributes to anxiety, while trauma-related disorders relate to a state of diminished mental health.^50^ The profound sense of dependence on external survival aids underscores their reliance on humanitarian assistance for basic needs, which compounds the emotional toll.^59^

The thoughts and transition from being self-sufficient to relying on assistance for basic needs lead to trauma, feelings of hopelessness and daily experiences of despair for some participants in our study. According to Vinck and colleagues in their study of gender-based violence among conflict-affected populations in the Democratic Republic of Congo, displaced people tend to lose their hope of living a normal life.^59^ Similarly, a study conducted in Ukraine revealed that conflicts or crises subject their victims to significant emotional toll and despair.^60^ The lack of control over one's life, coupled with uncertainties about what the future holds for them in terms of being free and independent again, creates significant emotional strain and feelings of hopelessness.^61^

Furthermore, as reported in our study, the absence of formal education, vocational training and livelihood support deprives displaced persons of critical avenues for self-determination, worsening their dependency and dampening morale. Previous studies indicate that victims of humanitarian crises, including those in Nigeria and other West African countries, face diverse challenges.^50,51^ Literature on protracted displacement has emphasized the psychological consequences of prolonged idleness, which include low self-efficacy and social disengagement.^62^ Importantly, the lack of opportunities for growth does not occur in a vacuum, it intersects with other dimensions of structural exclusion, including limited access to external networks and weak government accountability mechanisms. This suggests that solutions must extend beyond service provision toward creating enabling environments where displaced individuals can regain their dignity and agency. Programs that integrate education, skills-building and employment pathways, even in displacement contexts, are essential for both psychosocial and socioeconomic recovery.

Although internal social support exists, there were variances reported regarding external social networks. Scattered families and friends across vast distances make physical visits infrequent or even impossible, coupled with limited access to mobile phones or the internet, therefore leading to feelings of isolation and disinterest in social connections. An Iraqi study reported that IDPs are restricted from owning a mobile and have to rely heavily on a central calling station, which is sometimes overcrowded and thus limits their ability to communicate with the external community.^63^ By contrast, a study conducted in Afghanistan reported that the vast majority of youths in IDP camps have access to personal mobile phones, albeit the financial burden of purchasing access to the internet persists.^64^ A plausible exploration of social support as highlighted by previous studies revealed a range of complexities shaped by factors such as location and the length of displacement.^65^ Other factors include the accessibility of humanitarian assistance and political willpower.^53^

Despite the challenges, participants in our study lean on their friends and family for social support and relationships. These findings are consistent with another study in the literature that reported the importance of social support networks in mitigating the challenges faced by residents of IDP camps.^3^ According to Gichunge and colleagues in their study on predictors of social support, physical health and mental health among internally displaced persons in Kenya, those who had lived in the IDP camps for a decade leaned more on the existing social network and support for survival compared with their counterparts who were new in the camps.^65^ This social support among themselves not only helps them to survive the harsh challenges but also reinforces solidarities among them.^65^ This corroborates with a study in Cameroon, which revealed that solidarities among displaced persons promote community satisfaction and reinforce hope.^66^ Hence, while the fundamental importance of social support remains unchanged, it is crucial to consider contextual nuances. Tailored interventions must be designed to recognize and utilize both internal and external support systems to effectively address the complex needs of displaced populations in crises.^3^

### Strength and limitations of the study

In this study, we explored the lived experience of the displaced population in a camp in Cross River, Nigeria. Through a qualitative phenomenology analysis, this study provides a detailed understanding of participants' viewpoints, highlighting the daily challenges they face and the intricate relationship between socioenvironmental factors and psychosocial well-being. This study contributes to the expanding body of research on the experiences of displaced populations in crisis settings. Findings from this study provide valuable insights to support targeted interventions and support systems tailored to the needs of displaced individuals.

Despite the strength of our study, we also acknowledge some limitations. First, our study was conducted among one IDP camp in Nigeria. Hence, participants were selected from a specific location, which potentially may have limited the generalizability of our findings. Additionally, due to the cross-sectional nature of our study, it was difficult to capture how their experience changes over time and comprehend the evolving nature of displacement experiences. Future research should address these limitations to enhance the validity and applicability of findings related to displaced populations in crisis settings.

### Conclusion

Based on the comprehensive discussion provided, the conclusion of the study underscores the multifaceted challenges faced by displaced individuals living in an IDP camp, emphasizing the harsh physical environment, the psychological toll of displacement and the critical importance of access to basic necessities and social support networks. However, while there are broad consistencies, nuanced differences exist across regions, influenced by sociopolitical dynamics, cultural norms and the efficacy of humanitarian aid. These variations underscore the need for context-specific interventions that recognize and address the diverse needs of displaced populations. Additionally, our study also emphasized the importance of establishing an accessible social support system for IDPs to connect with external communities. This connection may enhance their hope for a normal life, build resilience and facilitate their social integration back into society.

## Data Availability

All data relevant to the study are included in the article or uploaded as supplementary information.
